# Landscape of Statin as a Cornerstone in Atherosclerotic Cardiovascular Disease

**DOI:** 10.31083/j.rcm2412373

**Published:** 2023-12-29

**Authors:** Cheng Yang, Yong-Jian Wu, Jie Qian, Jian-Jun Li

**Affiliations:** ^1^State Key Laboratory of Cardiovascular Disease, Fuwai Hospital, National Center for Cardiovascular Diseases, Chinese Academy of Medical Sciences and Peking Union Medical College, 100037 Beijing, China

**Keywords:** statins, cardiovascular disease, lipid-lowering, cornerstone

## Abstract

Atherosclerosis, the key pathogenesis of cardiovascular disease, is a leading cause of death and disability worldwide. Statins are first-line lipid-lowering drugs, which have been demonstrated to be powerful agents for anti-atherosclerosis. Numerous studies have confirmed the cardiovascular benefits and long-term safety of statins in a wide range of patients. Statins play an indispensable and irreplaceable part in the prevention and treatment of atherosclerotic cardiovascular disease (ASCVD). In this article, we summarize the evolution of statins and their role in the treatment of cholesterol. The anti-atherosclerotic mechanism of statins, its efficacy, safety and clinical outcomes in secondary and primary prevention of ACSVD in different patient populations, the combination treatment effects, and guideline recommendations are also detailed. This paper highlights the profound significance of statins as the most successful anti-atherogenic drug in the cardiovascular field.

## 1. Introduction

**Fig. 1.**
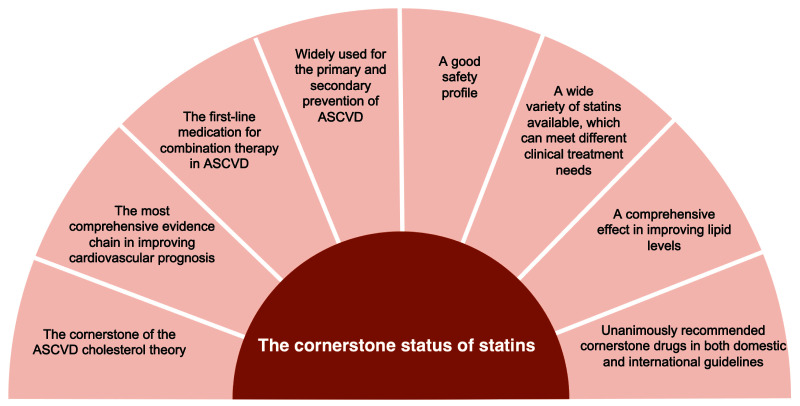
**Reasons for statin as a cornerstone in atherosclerotic cardiovascular disease**. ASCVD, atherosclerotic cardiovascular disease.

**Fig. 2.**
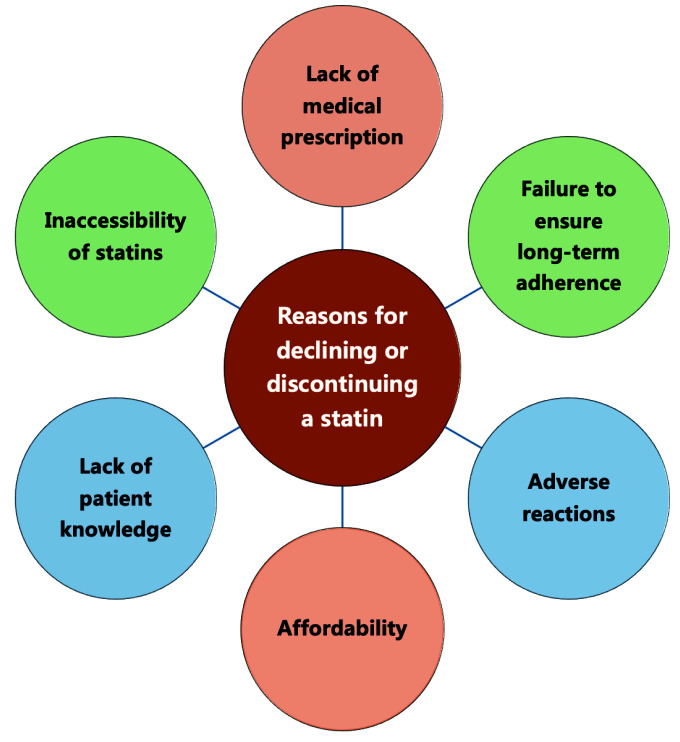
**Reasons for declining or discontinuing a statin**.

Cardiovascular disease (CVD) remains a leading cause of death and disability world-wide, contributing significantly to rising medical expenses. Atherosclerosis is the pathological foundation for CVD, with dyslipidemia, especially low-density lipoprotein cholesterol (LDL-C), as its key risk factor [1]. Statins, pivotal in CVD management, effectively reduce blood lipid levels, primarily LDL-C [1]. This action slows atherosclerosis progression and diminishes the likelihood of cardiovascular events and fatalities [1]. Statins are the primary drugs recommended by society guidelines for lipid-lowering therapy (LLT) and anti-atherosclerotic cardiovascular disease (anti-ASCVD) [1]. Even with the emergence of various new lipid-lowering drugs, the status of statins as the cornerstone for anti-ASCVD remains unchanged (Fig. 1) [1]. However, in clinical practice, there are still a significant number of patients who decline or discontinue statins [1, 2]. This may be attributable to lack of medical prescriptions, patients’ fear of adverse reactions, and failure to ensure long-term adherence (Fig. 2) [1, 2]. This paper studies the clinical benefits of statins from various aspects by reviewing the development and evidence-based history of statins, and demonstrating the important role of statins in the prevention and treatment of ASCVD in clinical practice.

## 2. A Historical Review of Statins

**Fig. 3.**
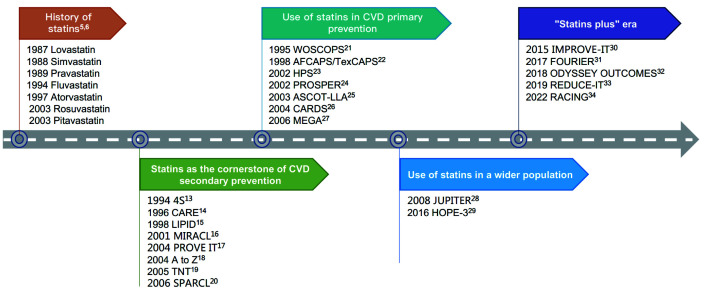
**Discovery and evidence-based history of statins**. CVD, cardiovascular disease; REDUCE-IT, reduction of cardiovascular events with icosapent ethyl–intervention trial; 4S, scandinavian simvastatin survival Study; CARE, cholesterol and recurrent events; LIPID, long-term intervention with pravastatin in ischaemic disease; MIRACL, myocardial ischemia reduction with aggressive cholesterol lowering; PROVE IT, pravastatin or atorvastatin evaluation and infection therapy; TNT, treating to new targets; SPARCL, stroke prevention by aggressive reduction in cholesterol levels; WOSCOPS, west of scotland coronary prevention study; AFCAPS/TexCAPS, air force/texas coronary atherosclerosis prevention study; HPS, heart protection study; PROSPER, pravastatin in elderly individuals at risk of vascular disease; ASCOT-LLA, anglo-scandinavian cardiac outcomes trial; CARDS, collaborative atorvastatin diabetes study; MEGA, management of elevated cholesterol in the primary prevention group of adult japanese; JUPITER, justification for the use of statins in prevention: an intervention trial evaluating rosuvastatin; HOPE-3, heart outcomes prevention evaluation-3; ODYSSEY OUTCOMES, evaluation of cardiovascular outcomes after an acute coronary syndrome during treatment with alirocumab; IMPROVE-IT, improved reduction of outcomes: vytorin efficacy international trial; FOURIER, further cardiovascular outcomes research with PCSK9 inhibition in subjects with elevated risk; REDUCE-IT, reduction of cardiovascular events with icosapent ethyl–intervention trial; RACING, long-term efficacy and safety of moderate-intensity statin with ezetimibe combination therapy versus high-intensity statin monotherapy in patients with atherosclerotic cardiovascular disease.

In the 1950s and 1960s, researchers identified 3-hydroxy-3-methyl-glutaryl-CoA (HMG-CoA) reductase as the key rate-limiting enzyme for cholesterol synthesis and regulation in the human body [3]. In 1973, after screening thousands of molds, Akira Endo’s team found mevastatin, the first natural HMG-CoA reductase inhibitor, from the culture medium of Penicillium citrinum [3]. Mevastatin has a similar structure to HMG-CoA and can competitively inhibit HMG-CoA reductase [3]. However, the research and development of mevastatin was terminated due to the increased risk of developing malignant tumors [3]. During the same period, both Akira Endo’s team and Merck’s team independently discovered lovastatin from another mold [4]. In 1987, lovastatin was approved by the U.S. Food and Drug Administration (FDA), making it the first commercial statin [3]. Since then, a series of statins have entered the market, which marked the beginning of a legendary journey of statins and revolutionized cholesterol management and CVD prevention (Fig. 3) [5, 6].

## 3. Statins as Cornerstone Drugs for Lowering Cholesterol

The understanding of atherosclerosis underwent a significant shift in 1913, as Nikolai N. Anitschkow demonstrated that diets high in cholesterol could induce atherosclerotic lesions in rabbit models, linking atherosclerosis to blood cholesterol levels [7]. This discovery transformed the widely held belief that atherosclerosis was caused by aging and ushered in a new era for atherosclerosis research. Since 1960s, a series of epidemiological and dietary interventional studies revealed a direct correlation between coronary heart disease (CHD) and cholesterol [8, 9, 10, 11, 12]. Furthermore, these studies illustrated that lowering cholesterol through dietary intervention can reduce CHD risk [8, 9, 10, 11, 12]. The American Heart Association (AHA) embraced the concept that cholesterol is the cause of atherosclerosis in 1961, and called for high-risk groups to change their diet.

Despite mounting evidence for the cholesterol hypothesis, it was not accepted by all experts. Specifically, Oliver and colleagues—in the middle and late 20th century—doubted that high cholesterol was the key factor of CHD, as well as the role of reducing lipid concentrations in atherosclerosis management [7]. With the advent of statins and the emergence of a large number of cardiovascular outcome studies, lowering LDL-C has been demonstrated to be effective in significantly reducing the risk of cardiovascular events and deaths, while early and intensive LDL-C lowering could bring even more benefits to the cardiovascular system (Table 1, Ref. [13, 14, 15, 16, 17, 18, 19, 20, 21, 22, 23, 24, 25, 26, 27, 28, 29, 30, 31, 32, 33, 34, 35, 36, 37, 38, 39, 40, 41, 42, 43, 44], Fig. 3). The latest meta-analysis revealed that statins could reduce all-cause mortality by 9%, myocardial infarction (MI) by 29% and stroke by 14% [45]. These studies have elevated the cholesterol hypothesis to new heights.

**Table 1.** **Classic RCTs on statins**.

Trial	Year of release	Participants characteristics	Participants	Intervention methods	Follow-up period	Main results
Primary prevention						
WOSCOPS [21]	1995	-	6595 men without MI history	pravastain 40 mg/d VS placebo	4.9 years of mean follow-up	31% reduction in the risk of death from nonfatal MI or CHD; 32% reduction in the risk of CVD deaths; 22% reduction in the risk of all-cause mortality.
AFCAPS/TexCAPS [22]	1998	-	5608 men and 997 women without clinical ASCVD	lovastatin 20–40 mg/d VS placebo	5.2 years of mean follow-up	37% reduction in the risk of the first ACS; 40% reduction in MI risk; 33% reduction in coronary revascularization risk; 32% reduction in UA risk; 25% reduction in the risk of cardiovascular events.
ALLHAT- LLT [35]	2002	hypertension	10,355 hypertensive patients aged 55 years or older	pravastatin 40 mg/d VS conventional therapy	4.8 years of mean follow-up	9% reduction in the risk of CHD events (no statistically significant difference).
ASCOT-LLA [25]	2003	hypertension	10,305 hypertensive patients with no history of CAD	atorvastatin 10 mg/d VS placebo	3.3 years of median follow-up	36% reduction in CHD deaths and nonfatal MI risk; 29% reduction in the risk of total coronary events; 27% reduction in stroke risk.
CARDS [26]	2004	diabetes	2838 patients with type 2 diabetes	atorvastatin 10 mg/d VS placebo	3.9 years of median follow-up	37% reduction in the risk of combined endpoints (acute CHD events, coronary revascularization; stroke); 27% reduction in all-cause mortality; 36% reduction in the risk of acute CHD events; 36% reduction in the risk of acute coronary events; 48% reduction in stroke risk.
MEGA [27]	2006	hypercholesterolemia	3966 hypercholesterolemia patients	diet plus pravastatin 80 mg/d VS diet	5.3 years of mean follow-up	33% reduction in the risk of first CHD; 48% reduction in MI risk; 26% reduction in cardiovascular events risk.
JUPITER [28]	2008	CRP elevation	17,802 patients, CRP >2.0 mg/L, without CVD or diabetes history	rosuvastatin 20 mg/d VS placebo	1.9 years of median follow-up	44% reduction in the risk of combined endpoints (MI, stroke, arterial revascularization, UA hospitalization or cardiac deaths); 54% reduction in MI risk; 48% reduction in stroke risk; 20% reduction in all-cause mortality.
SHARP [43]	2011	chronic kidney disease	9270 patients with chronic kidney disease	simvastatin 20 mg/d plus ezetimibe 10 mg/d VS placebo	4.9 years of median follow-up	17% reduction in major atherosclerotic events; 25% reduction in the risk of non-hemorrhagic stroke; 21% reduction in the risk of arterial revascularization.
HOPE-3 [29]	2016	-	12,705 intermediate-risk CVD patients	rosuvastatin 10 mg/d VS placebo	5.6 years of median follow-up	24% reduction in the risk of combined endpoints (cardiovascular death, nonfatal MI or nonfatal stroke); 35% reduction in. MI risk; 30% reduction in stroke risk; 32% reduction in revascularization risk.
Secondary prevention						
4S [13]	1994	CHD	4444 patients with angina or previous MI	simvastatin 20–40 mg/d VS placebo	5.4 years of median follow-up	30% reduction in all-cause mortality; 37% reduction in the risk of myocardial revascularization; 42% reduction in the risk of coronary deaths; 42% reduction in the risk of major coronary events; 30% reduction in stroke risk.
CARE [14]	1996	CHD	3583 men and 576 women with MI	pravastatin 40 mg/d VS placebo	5.0 years of median follow-up	24% reduction in CHD deaths or nonfatal MI risk; 23% reduction in nonfatal MI risk; 23% reduction in the risk of CABG or PTCA; 31% reduction in stroke risk.
LIPID [15]	1998	CHD	9014 patients with MI or UA history	pravastatin 40 mg/d VS placebo	6.1 years of median follow-up	24% reduction in the risk of CHD deaths; 25% reduction in the risk of CVD deaths; 22% reduction in the risk of all-cause mortality; 19% reduction in stroke risk; 24% reduction in the risk of fatal CHD or nonfatal MI; 29% reduction in MI risk.
LIPS [36]	2002	CHD	1667 patients aged 18–80 after CAD angioplasty	fluvastatin 40 mg/d VS placebo	3.9 years of median follow-up	22% reduction in MACE risk (cardiac death, nonfatal MI or surgical reintervention).
GREACE [38]	2002	CHD	1600 CHD patients	atorvastatin 10–80 mg/d VS conventional therapy	3.0 years of mean follow-up	51% reduction in CHD recurrence or death; 43% reduction in all-cause mortality; 47% reduction in coronary death risk; 47% reduction in stroke risk.
PACT [39]	2004	CHD	3408 CHD patients	pravastatin 20–40 mg/d VS placebo	4 weeks	11.6% of combined endpoints (death, MI recurrence or UA readmission) VS 12.4% (no statistically significant difference).
ALLIANCE [40]	2004	CHD	2442 CHD patients	atorvastatin 10–80 mg/d VS conventional therapy	51.5 months of mean follow-up	17% reduction in the risk of combined endpoints (cardiac death, nonfatal MI, cardiac arrest resuscitation, cardiac revascularization and UA re-admission).
TNT [19]	2005	CHD	10,001 CHD patients	atorvastatin 80 mg/d VS 10 mg/d	4.9 years of median follow-up	22% reduction in the risk of combined endpoints (CHD death, nonfatal and nonsurgical MI, cardiac arrest resuscitation and stroke); 22% reduction in the risk of nonfatal and nonsurgical MI; 25% reduction in stroke risk.
CCSPS [41]	2005	CHD	4870 MI patients	Xuezhikang 0.6 g/bid VS placebo	4 years of mean follow-up	45% reduction in the risk of CHD events; 31% reduction in the risk of CHD deaths; 33% reduction in the need for PCI and(or) CABG; 33% reduction in all-cause mortality.
REAL-CAD [44]	2018	CHD	13,054 CHD patients	pitavastatin 4 mg/d VS 1 mg/d	3.9 years of median follow-up	19% reduction in the risk of combined endpoints (cardiovascular deaths, nonfatal MI, nonfatal stroke or UA emergency hospitalization); 43% reduction in MI risk; 19% reduction in all-cause mortality risk; 22% reduction in the risk of cardiovascular deaths; 14% reduction in the risk of coronary revascularization.
MIRACL [16]	2001	ACS	3086 ACS patients	atorvastatin 80 mg/d VS placebo	16 weeks	16% relative reduction in the risk of primary combined endpoint deaths, nonfatal AMI, cardiac arrest with resuscitation, or myocardial ischemia with rehospitalization; 26% reduction in ischemia with objective evidence and emergency rehospitalization risk; 50% reduction in stroke risk.
FLORIDA [37]	2002	ACS	540 AMI patients	fluvastatin 40 mg/d VS placebo	12 months	2.6% all-cause mortality VS 4.0% (no statistically significant difference).
PROVE IT [17]	2004	ACS	4162 ACS patients	atorvastatin 80 mg/d VS pravastatin 40 mg/d	2.0 years of median follow-up	16% reduction in the risk of combined endpoints (all-cause mortality, MI and UA re-admission, revascularization and stroke after at least 30 days at random); 14% reduction in CHD deaths, MI or revascularization risk; 29% reduction in the risk of UA recurrence.
A to Z [18]	2004	ACS	4497 ACS patients	simvastatin 40 mg/d for 1 month followed by 80 mg/d VS placebo for 4 months followed by simvastatin 20 mg/d	6–24 months	25% reduction in the risk of main endpoints (cardiac death, nonfatal MI, ACS re-admission and stroke) four months until the end of follow-up period.
IDEAL [42]	2005	ACS	8888 patients with AMI history	atorvastatin 80 mg/d VS 20–40 mg/d	4.8 years of median follow-up	13% reduction in the risk of combined endpoints (CHD death, nonfatal AMI, cardiac arrest resuscitation); 17% reduction in nonfatal MI risk; 23% reduction in the risk of revascularization; 24% reduction in PAD risk.
IMPROVE IT [30]	2015	ACS	18,144 ACS patients	simvastatin 40 mg/d plus ezetimibe 10 mg/d VS simvastatin 40 mg/d	6 years of median follow-up	6.4% reduction in the risk of combined endpoints (cardiovascular deaths, nonfatal MI, UA hospitalization, coronary revascularization, nonfatal stroke); 13% reduction in MI risk; 14% reduction in stroke risk.
ODYSSEY OUTCOMES [32]	2018	ACS	18,924 patients with recent ACS	high-intensity statin plus alirocumab VS high-intensity statin	2.8 years of median follow-up	15% reduction in the risk of combined endpoints (CHD deaths, nonfatal MI, ischemic stroke, UA hospitalization); 15% reduction in the risk of all-cause mortality; 14% reduction in the risk of nonfatal MI; 39% reduction in UA hospitalization risk; 27% reduction in ischemic stroke.
SPARCL [20]	2006	stroke/TIA	4731 stroke/TIA patients	atorvastatin 80 mg/d VS placebo	4.9 years of median follow-up	16% reduction in the risk of first stroke recurrence; 20% reduction in the risk of MACE.
FOURIER [31]	2017	ASCVD	27,564 ASCVD patients	high-intensity or moderate-intensity statin plus evolocumab VS high-intensity or moderate-intensity statin	2.2 years of median follow-up	15% reduction in the risk of combined endpoints (cardiovascular deaths, MI, stroke, UA hospitalization or coronary revascularization); 27% reduction in MI risk; 21% reduction in stroke risk; 22% reduction in the risk of coronary revascularization.
RACING [34]	2022	ASCVD	3780 ASCVD patients	rosuvastatin 10 mg/d plus ezetimibe 10 mg/d VS rosuvastatin 20 mg/d	3 years of median follow-up	No significant difference in the main endpoints (CHD deaths, MACE, nonfatal stroke) and the incidence of each group.
Primary/secondary prevention						
HPS [23]	2002	-	20,536 patients with CHD, other occlusive arterial disease or diabetes	simvastatin 40 mg/d VS placebo	5.0 years of median follow-up	18% reduction in coronary death risk; 13% reduction in all-cause mortality; 27% reduction in major coronary events risk; 25% reduction in stroke risk; 24% reduction in revascularization risk.
PROSPER [24]	2002	-	5804 patients aged 70–82 with a history of, or risk factors for, vascular disease	pravastatin 40 mg/d VS placebo	3.2 years of mean follow-up	15% reduction in combined endpoint risk of CHD deaths, nonfatal MI and stroke; 19% reduction in CHD deaths and nonfatal MI risk; 25% reduction in TIA risk.
REDUCE-IT [33]	2019	TG elevation	8179 patients with a fasting TG level of 135–499 mg/dL	statins plus IPE VS placebo	4.9 years of median follow-up	25% reduction in the risk of combined endpoints (CHD deaths, nonfatal stroke, coronary revascularization or UA); 20% reduction in the risk of CHD deaths; 31% reduction in MI risk; 28% reduction in stroke risk.

ASCVD, atherosclerotic cardiovascular disease; ACS, acute coronary syndrome; AMI, acute myocardial infarction; CABG, coronary artery bypass graft; CAD, coronary artery disease; CHD, coronary heart disease; MACE, major adverse cardiac event; MI, myocardial infarction; PCI, percutaneous coronary intervention; PTCA, percutaneous transluminal coronary angioplasty; RCT, randomized controlled trials; UA, unstable angina; TG, triglyceride; TIA, transient ischemic attack; CRP, C-reactive protein; PAD, peripheral arterial disease; CVD, cardiovascular disease; HPS, heart protection study; 4S, scandinavian simvastatin survival study.

In 2017, the European Atherosclerosis Society (EAS) issued the Consensus Statement on the causality of LDL and ASCVD, and conducted a meta-analysis of more than 200 trials, including genetics, epidemiology and randomized controlled trials (RCT) [46]. It demonstrated a remarkably consistent dose-dependent log-linear association between the absolute magnitude of exposure of the vasculature to LDL-C and the risk of ASCVD; ASCVD risk would increase with the increase of LDL-C exposure, while lowering LDL-C could reduce ASCVD risks proportionally [46]. This consensus statement recognized the causality between LDL-C and ASCVD, and the cholesterol hypothesis became the cholesterol theory [46]. At present, many blood lipid guidelines regard LDL-C as the primary target for ASCVD prevention and treatment.

## 4. Statins as Cornerstone Drugs for Anti-atherosclerosis

**Fig. 4.**
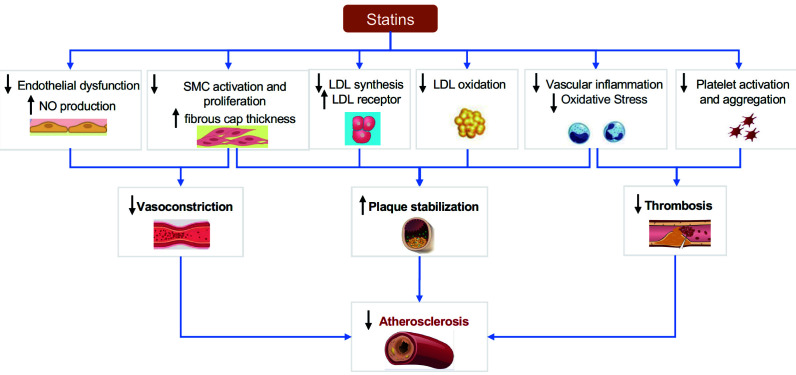
**Antiatherogenic mechanisms of statins**. NO, nitric oxide; SMC, smooth muscle cells; LDL, low-density lipoprotein.

Over time, we have gained a deeper understanding of atherosclerosis. Its pathogenesis involves not only cholesterol deposition but also endothelial dysfunction, inflammation, oxidative stress, and smooth muscle cell proliferation [47, 48]. The role of statins in preventing the progression of atherosclerosis has been demonstrated in studies such as Post-Coronary Artery Bypass Graft (POST-CABG) [49], Reversal of Atherosclerosis with Aggressive Lipid Lowering (REVERSAL) [50], A Study To Evaluate the effect of Rosuvastatin On Intravascular ultrasound-Derived coronary atheroma burden (ASTEROID) [51], Study of Coronary Atheroma by Intravascular Ultrasound: Effect of Rosuvastatin vs. Atorvastatin (SATURN) [52], and Measuring Effects on Intima-Media Thickness: An Evaluation of Rosuvastatin (METEOR-China) [53]. In recent years, research has demonstrated that in addition to lowering cholesterol, statins have anti-atherosclerotic effects through other pathways (Fig. 4) [54, 55].

### 4.1 Lowering Cholesterol

Statins competitively inhibit HMG-CoA reductase and block the intracellular pathway of mevalonic acid metabolism [56]. The ultimate dual effect is reducing cholesterol synthesis and increasing the clearance by stimulating feedback to upregulate LDL receptors on the surface of liver cells [56].

### 4.2 Protecting the Endothelium

Endothelial dysfunction is one of the early manifestations of atherosclerosis, and is characterized by reduced synthesis of endothelial nitric oxide synthase (eNOS) and decreased bioavailability of nitric oxide (NO) [57]. Statins improve endothelial function in patients with atherosclerosis through both cholesterol-dependent and cholesterol-independent pathways [57]. The former has been demonstrated in LDL-C monotherapy studies [58]. While the specific mechanisms of the latter are still unclear, it is known to involve increased stability of eNOS mRNA through the Rho/ROCK pathway, activation of eNOS by serine-threonine protein kinase Akt, and modulation of eNOS activity by caveolin-1 [57]. Furthermore, recent studies have shown that statins could improve endothelial function via suppression of epigenetic-driven EndMT [59].

### 4.3 Reducing Inflammation and Oxidative Stress

Atherosclerosis is a chronic inflammatory disease characterized by the activation of pro-inflammatory signaling pathways, expression of cytokines/chemokines, and increased oxidative stress [60]. Studies such as Canakinumab Antiinflammatory Thrombosis Outcome Study (CANTOS) suggest that inflammation can be treated as another target in fighting atherosclerosis [61]. Statins can inhibit the migration and activation of inflammatory cells by reducing the expression of endothelial adhesion molecules, interferon-γ (INF-γ), tumor necrosis factor α (TNF-α), interleukin-1 (IL-1). Statins can also inhibit the generation of reactive oxygen species (ROS), reduce LDL oxidation and improve NO activity, thereby slowing the progression of atherosclerosis and contributing to plaque stability [55, 57, 62].

In addition, statins inhibit the proliferation of smooth muscle cells, which is associated with increased NO activity and Rho inhibition [57, 62]. It has been reported that statins can reduce platelet activation and thromboxane A2 synthesis [54, 62]. In summary, statins slow atherosclerosis through multiple pathways, which explains their pleiotropic clinical benefits in addition to cholesterol lowering.

## 5. Statins as Cornerstone Drugs Benefiting a Wide Range of Populations

**Fig. 5.**
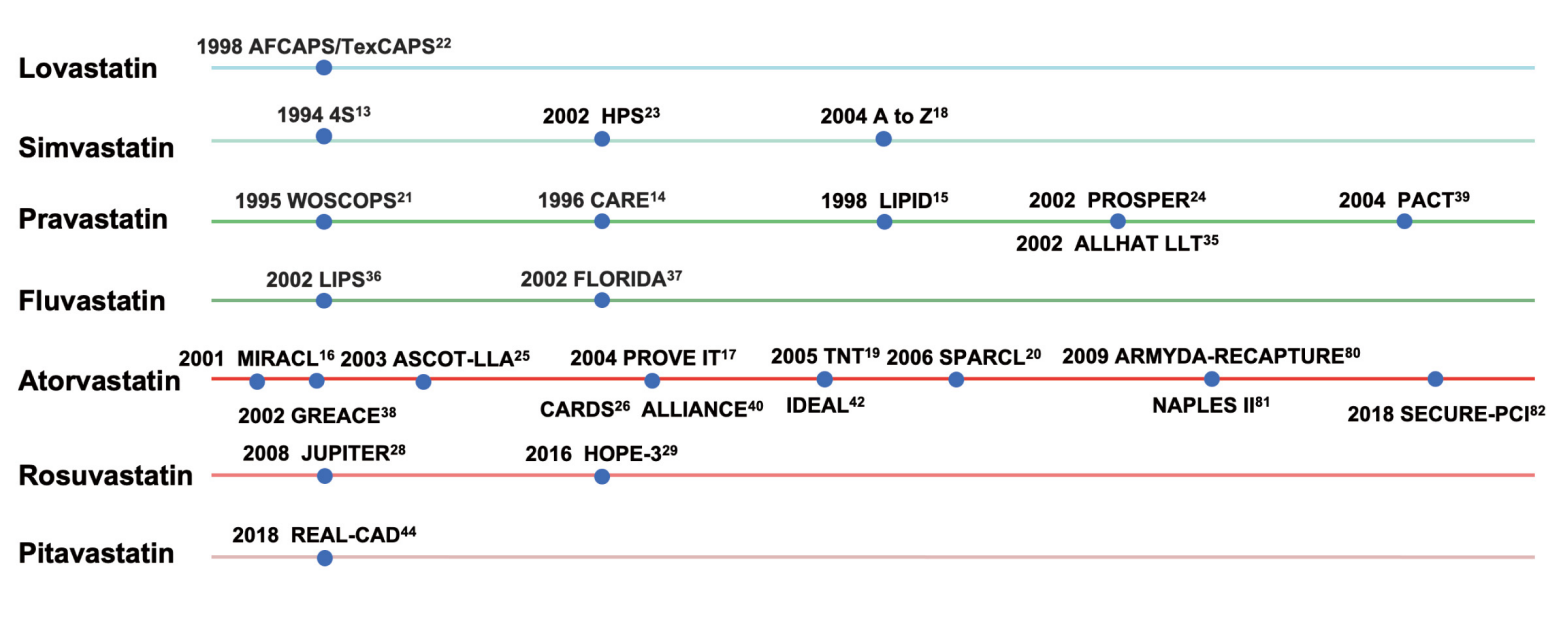
**Clinical studies of different types of statins**. AFCAPS/TexCAPS, air force/texas coronary atherosclerosis prevention study; 4S, scandinavian simvastatin survival study; HPS, heart protection study; WOSCOPS, west of scotland coronary prevention study; CARE, cholesterol and recurrent events; LIPID, long-term intervention with pravastatin in ischaemic disease; PROSPER, pravastatin in elderly individuals at risk of vascular disease; ALLHAT LLT, antihypertensive and lipid-lowering treatment to prevent heart attack trial; PACT, pravastatin in acute coronary treatment; LIPS, lescol intervention prevention study; FLORIDA, fLuvastatin on risk diminishing after acute myocardial infarction; MIRACL, myocardial ischemia reduction with aggressive cholesterol lowering; ASCOT-LLA, anglo-scandinavian cardiac outcomes trial; PROVE IT, pravastatin or atorvastatin evaluation and infection therapy; TNT, treating to new targets; SPARCL, stroke prevention by aggressive reduction in cholesterol levels; ARMYDA-RECAPTURE, atorvastatin for reduction of myocardial damage during angioplasty; GREACE, GREek atorvastatin and coronary-heart-disease evaluation; CARDS, collaborative atorvastatin diabetes study; ALLIANCE, aggressive lipid-lowering initiation abates new cardiac events; IDEAL, incremental decrease in end points through aggressive lipid lowering ; NAPLES II, novel approaches for preventing or limiting events II; SECURE-PCI, statins evaluation in coronary procedures and revascularization; JUPITER, justification for the use of statins in prevention: an intervention trial evaluating rosuvastatin; HOPE-3, heart outcomes prevention evaluation-3; REAL-CAD, randomized evaluation of aggressive or moderate lipid lowering therapy with pitavastatin in coronary artery disease.

Over the past 30 years, emerging RCTs, real-world studies, and meta-analyses have documented the benefits of statin therapy to the public. Statins have become the cornerstone in primary/secondary prevention of ASCVD, and play an irreplaceable role in this field (Table 1; Table 2, Ref. [45, 63, 64, 65, 66, 67, 68, 69, 70, 71, 72, 73, 74, 75, 76, 77, 78, 79] and Fig. 5) [13, 14, 15, 16, 17, 18, 19, 20, 21, 22, 23, 24, 25, 26, 27, 28, 29, 30, 31, 32, 33, 34, 35, 36, 37, 38, 39, 40, 41, 42, 43, 44, 46, 63, 64, 65, 66, 67, 68, 69, 70, 71, 72, 73, 74, 75, 76, 77, 78, 79, 80, 81, 82].

**Table 2.** **Classic meta-analyses on statins**.

Year of release	Journal	Trials included	Participants	Intervention methods	Main results
1999 [63]	JAMA	5 RCTs	primary/secondary prevention, 30,817 middle-aged and elderly patients	statin VS placebo	Statin therapy lowered the risk of major coronary events by 31% and all-cause mortality by 21%; The risk reduction was similar for men and women and for elderly and middle-aged persons.
2005 [64]	Lancet	14 RCTs	primary/secondary prevention, 90,056 patients	statin VS placebo	A reduction in LDL-C of 1 mmol/L by statin produced a 12% reduction in the risk of all-cause mortality, 19% in coronary deaths, 23% in coronary events and 17% in stroke.
2007 [65]	Heart	6 RCTs	110,271 CHD patients	high-intensity statin VS moderate-intensity statin	Intensive statin therapy reduced the risk of MACE by 16%, and admission to hospital for heart failure by 28%; For the ACS subgroup, intensive statin therapy reduced the risk of all-cause mortality by 25%; for the stable CHD subgroup there was has no significant effect.
2008 [66]	Lancet	14 RCTs	18,686 diabetic patients and 71,370 non-diabetic patients	stain VS placebo	A reduction in LDL-C of 1 mmol/L by statin produced a 9% reduction in the risk of all-cause mortality for diabetic patients and 13% reduction for non-diabetic patients; major vascular events reduced by 21% for both groups.
2009 [67]	Lancet Neurol	24 RCTs	primary/secondary stroke prevention, 165,792 patients	statin/strong statin VS placebo/weak statin	Statin/strong statin therapy reduced stroke risk by 18% (19% for primary prevention and 12% for secondary prevention).
2010 [68]	Lancet	26 RCTs	primary/secondary prevention, 169,138 patients	statin/strong statin VS placebo/weak statin	A reduction in LDL-C of 1 mmol/L by statin produced a 22% reduction in the risk of major vascular events, 10% in all-cause mortality, and 20% in CHD deaths; Intensive statin therapy reduced the risk of cardiovascular events to a larger extent.
2011 [69]	Eur Heart J	10 RCTs	41,778 CHD patients	strong statin VS weak statin	Intensive statin therapy reduced the risk of CHD deaths and nonfatal MI by 10%, fatal MI by 18% and stroke by 14%; In the ACS subgroup, intensive statin therapy reduced the risk of all-cause mortality by 25% and cardiovascular deaths by 26%.
2012 [70]	J Am Coll Cardiol	18 RCTs	primary/secondary prevention, 141,235 patients (40,275 women)	statin/strong statin VS placebo/weak statin	Statin/strong statin therapy significantly reduced the risk of cardiovascular events (19% for women and 23% for men); The benefit of statins was statistically significant in both sexes, regardless of the type of baseline risk, or type of endpoint and in both primary and secondary prevention.
2012 [71]	Lancet	27 RCTs	primary/secondary prevention, 174,149 patients	statin/strong statin VS placebo/weak statin	A reduction in LDL-C of 1 mmol/L by statin produced a 21% reduction in major vascular events; For all the five categories of baseline 5-year major vascular event risk (<5%, ≥5% to <10%, ≥10% to <20%, ≥20% to <30%, ≥30%), there was a significant decrease in the risk of major vascular events (by 38%, 31%, 21%, 19%, and 30%).
2014 [72]	Am J Cardiol	20 RCTs	8750 ACS patients before or after PCI	pre- and post-PCI statin administration/high statin doses VS no statin administration/low statin doses	In the statin group, 30-day treatment reduced MI risk by 33%, while pre-PCI statin administration produced a bigger reduction (62%) compared with post-PCI; The risk of MACE and MACCE in the statin group reduced by 54% and 18%, respectively.
2015 [73]	Lancet	27 RCTs	primary/secondary prevention, 174,149 patients	statin/strong statin VS placebo/weak statin	A reduction in LDL-C of 1 mmol/L by statin produced a 21% reduction in major vascular events (16% for women and 22% for men, with no significant gender difference); In the risk reduction of major coronary events, coronary revascularization and stroke, there was no significant difference between men and women.
2016 [74]	JAMA	19 RCTs	primary/prevention, 71,344 patients	statin/strong statin VS placebo/weak statin	Statin treatment reduced the risk of all-cause mortality by 14%, cardiovascular deaths by 31%, stroke by 29%, MI by 36% and combined cardiovascular endpoints by 30%; Relative benefits appeared consistent in demographic and clinical subgroups.
2019 [75]	Lancet	28 RCTs	primary/secondary prevention, 186,854 patients	statin/strong statin VS placebo/weak statin	A reduction in LDL-C of 1 mmol/L by statin produced a 21% reduction in major vascular events; There was a significant reduction in major vascular events in all age groups (55 years or younger, 56–60 years, 61–65 years, 66–70 years, 71–75 years, and older than 75 years), although proportional reductions in major vascular events diminished slightly with age.
2020 [76]	Lancet	29 RCTs	primary/secondary prevention, 244,090 patients (21,492 of them aged 75 or above)	statin/strong statin VS placebo/weak statin (24 trials); statin plus ezetimibe/PCSK9 inhibitor VS statin	A reduction in LDL-C of 1 mmol/L by statin produced a significant decrease in major vascular events risk (26% for patients aged 75 or above, and 15% for patients below 15%, no statistically significant difference); For patients aged 75 or above, the risk of cardiovascular deaths, MI, stroke and coronary revascularization dropped by 15%, 20%, 27% and 20% with every 1 mmol/L reduction of LDL-C.
2021 [77]	JAMA Intern Med	8 RCTs	primary prevention, 65,383 patients aged 50–75	statin/strong statin VS placebo/weak statin	Treating 100 adults without known cardiovascular disease with a statin for 2.5 years prevented 1 MACE in 1 adult.
2022 [78]	JAMA Neurol	11 RCTs	20,163 patients with stroke	intensive VS less intensive LDL-C–lowering statin-based therapies	More intensive LDL-C–lowering statin-based therapies were associated with a reduced risk of recurrent stroke compared with less intensive ones (absolute risk, 8.1% vs 9.3%; relative risk, 12%).
2022 [79]	JAMA	23 RCTs, 3 observational studies	primary prevention, 513,291 patients	statin/strong statin VS placebo/weak statin	Statin treatment reduced the risk of cardiovascular combined endpoints by 28%, and the risk of MI, stroke and all-cause mortality by 33%, 22% and 8%, respectively.
2022 [45]	JAMA Intern Med	21 RCTs	primary prevention, >66,000 patients	statin/strong statin VS placebo/weak statin	Statin treatment reduced the absolute risk of all-cause mortality by 0.8% and the relative risk by 9%; The absolute risk of MI reduced by 1.3% and the relative risk by 29%; The absolute risk of stroke reduced by 0.4% and the relative risk by 14%.

ACS, acute coronary syndrome; CHD, coronary heart disease; LDL-C, low-density lipoprotein cholesterol; MACE, major cardiovascular adverse events; MACCE, major cardiovascular and cerebrovascular adverse events; MI, myocardial infarction; PCI, percutaneous coronary intervention; RCT, randomized controlled trial; PCSK9, proprotein convertase subtilisin/kexin type 9.

### 5.1 Statins in Secondary Prevention of ASCVD

#### 5.1.1 Patients with CHD

There is accumulating evidence for the use of statins in patients with CHD. Many RCTs have demonstrated the clinical benefits of statins in patients in different stages and different types of CHD [13, 14, 15]. As a result, the benefits of statin therapy have been extended from stable CHD to acute coronary syndromes (ACS), and from conservative drug treatment to surgical and interventional therapy [13, 14, 15]. The Scandinavian Simvastatin Survival Study (4S) [13] study is the first to confirm that statins can reduce cardiovascular events and all-cause mortality by reducing cholesterol levels. The 4S [13] and later Cholesterol and Recurrent Events (CARE) [14] and Long-Term Intervention with Pravastatin in Ischaemic Disease (LIPID) [15] studies confirmed the benefits of statins in the population with stable coronary artery disease, with or without elevated cholesterol levels. The China coronary secondary prevention study (CCSPS) [41] study design was similar to CARE [14], and the clinical results were similar, confirming the benefit of statins in the Chinese population. Myocardial Ischemia Reduction with Aggressive Cholesterol Lowering (MIRACL) [16], A to Z [18] and other trials focused on certain ACS patients and found that statin therapy significantly reduced major cardiovascular events (MACE). For patients undergoing percutaneous coronary intervention (PCI) or coronary artery bypass grafting (CABG), the risk MACE was significantly reduced by statins [36, 83].

Intensive statin therapy brings even greater benefits to people with CHD, which has been demonstrated in RCTs [17, 18, 19] and real-world studies [84, 85, 86]. Pravastatin or Atorvastatin Evaluation and Infection Therapy (PROVE IT) [17] is the first large-scale RCT to explore the effects of different types and intensities of statins on cardiovascular outcomes, which confirmed the benefits of early intensive lipid lowering, and together with subsequent studies, promoted lower LDL-C target values in the guidelines.

#### 5.1.2 Patients with Ischemic Stroke/Transient Ischemic Attack (TIA)

The use of statins also has an important role in secondary prevention and the acute phase of strokes [20, 87, 88, 89]. In the stratified analysis of Heart Protection Study (HPS), there was a highly significant 20% reduction in the rate of any major vascular event among patients with pre-existing cerebrovascular disease [87]. SPARCL, a secondary prevention study on patients with ischemic stroke/TIA, demonstrated that statins could reduce the risk of the first recurrence of stroke and the overall risk of cardiovascular and cerebrovascular events [20, 88, 89]. The greater the decrease of LDL-C, the lower the risk of such events [20, 88, 89]. Additionally, a meta-analysis demonstrated that antecedent use of statins was associated with improved outcomes in patients with acute ischemic stroke [90]. A recent meta-analysis also proved that statin therapy was useful for secondary prevention of stroke [91].

For patients with acute ischemic stroke, whether statins have been administered or not, the use of statins during hospitalization can improve the prognosis, and the earlier statins are introduced, the better the prognosis [92]. Even if patients have received intravenous thrombolysis (IVT) or intravascular treatment, the risk of death can still be reduced by statins [93, 94]. Moreover, “*in vivo*” pretreatment with statins in patients with first-time ischemic strokes was associated with better early outcome with decreased mortality during hospitalization and neurological disability at hospital discharge [95].

Although the pathophysiology, prognosis and clinical characteristics of patients with lacunar strokes are different from those of other acute cerebrovascular diseases, several observational studies have shown that the use of statins could reduce the risk of new cerebrovascular events in patients with small vessel disease [96]. Additional randomized controlled trials are necessary to determine whether statins contribute to the secondary prevention of lacunar infarcts.

#### 5.1.3 Patients with Peripheral Arterial Disease

Statin therapy is an important intervention in peripheral arterial disease (PAD) patients, which can lower the risk of all-cause mortality, MACE, and amputation [97]. The benefits of intensive statin therapy to further lower LDL levels are even greater [97]. Statin therapy also decreases MACE to PAD patients who have undergone revascularization [98].

### 5.2 Statins in Primary Prevention of ASCVD

#### 5.2.1 Hypercholesterolemia

For patients with hypercholesterolemia, studies such as West of Scotland Coronary Prevention Study (WOSCOPS) [21] and Air Force/Texas Coronary Atherosclerosis Prevention Study (AFCAPS/TexCAPS) [22] demonstrated that statins could significantly lower their risk of cardiovascular events. These studies mainly focused on Western populations, while Management of Elevated Cholesterol in the Primary Prevention Group of Adult Japanese (MEGA) [27] studied Japanese adults, providing further evidence for the benefits of statins for primary prevention in Asian populations.

#### 5.2.2 Coexistence of Hypertension and Dyslipidemia

Patients with hypertension are a major population for anti-ASCVD therapy. Lowering both LDL-C level and blood pressure is the cornerstone of ASCVD prevention and treatment. Three interventional studies on primary prevention of blood lipid among hypertensive patients, the subgroup analyses of Anglo-Scandinavian Cardiac Outcomes Trial–Lipid Lowering Arm (ASCOT-LLA) and Heart Outcomes Prevention Evaluation-3 (HOPE-3), all showed that statins bring significant cardiovascular benefits in addition to strict blood pressure control [25, 99]. Antihypertensive and Lipid-Lowering Treatment to Prevent Heart Attack Trial (ALLHAT-LLT) did not produce positive results, which might be related to the fact that 30% of patients in the routine intervention (control) group took statins, so that there was no major difference in cholesterol levels between the two groups [35]. Subgroup analyses of other studies, such as HPS and LIPID, also provided evidence for the benefits of statins in hypertensive patients [100].

#### 5.2.3 Coexistence of Diabetes and Dyslipidemia

The risk of occurrence and death of ASCVD is much higher for patients with diabetes [101]. Some studies suggest that dyslipidemia has the greatest impact on ASCVD risk among diabetic patients, highlighting the importance of manage their LDL-C levels [101]. The Collaborative Atorvastatin Diabetes Study (CARDS) and the HPS subgroup analyses showed that lowering LDL-C by statins could substantially reduce the risk of cardiovascular events [26, 102]. This is consistent with the results of a meta-analysis involving a large number of diabetic patients [103].

#### 5.2.4 Low-risk and Moderate-risk Populations

Other target populations of primary prevention can also benefit from statin therapy. As evidenced by Justification for the Use of Statins in Prevention: an Intervention Trial Evaluating Rosuvastatin (JUPITER) [28] and HOPE-3 [29], statins have cardiovascular benefits in patients with low ASCVD risk. Participants in the JUPITER study had high-sensitivity C-reactive protein (hs-CRP) but low blood lipid levels [28]. After lowering their LDL-C to the level recommended by current guidelines, the risk of cardiovascular events and deaths was significantly reduced, suggesting that patients with only increased level of inflammatory biomarkers could also benefit from statin therapy [28]. The HOPE-3 trial, which enrolled patents with an annual risk of major cardiovascular events of approximately 1% without increased LDL-C levels, also demonstrated the cardiovascular benefits of statins in groups of patients with only intermediate-risks [29]. The US Preventive Services Task Force (USPSTF) a systematic review on a large number of primary prevention studies of CVD, showed that statin therapy could reduce the risk of MI, stroke and all-cause mortality by 33%, 22% and 8% respectively [79].

### 5.3 Use of Statins in Other Populations

With the rapid rise in the prescription of statins for the low-risk and moderate-risk populations, there is now considerable experience in the use of statins across different age groups, non-ASCVD populations, and high-risk populations.

#### 5.3.1 Older Adults

Older adults can also benefit from statin therapy. The PROSPER study showed that for patients aged 70–82 years old, regardless of the presence of baseline coronary heart disease or the level of LDL-C, the risk of cardiovascular events was significantly reduced by statins [24]. A survey conducted in Korea on patients 65 years or older without CVD (n = 1,391,616) showed that statin use was significantly associated with a decrease in overall mortality risk after an average follow-up of 7.55 years [104]. A meta-analysis also confirmed that statin use in the primary prevention of CVD in elderly patients significantly reduces the risk of MI, stroke, and death [105].

#### 5.3.2 Children and Adolescents

Several studies have demonstrated the benefits of statins for children [106, 107, 108]. A Cochrane systematic review assessed the effectiveness and safety of statin treatment for heterozygous familial hypercholesterolemia (HeFH) in children aged 6–17, including 26 studies with a total of 1177 patients, and showed that statins could effectively reduce LDL-C [106]. Other studies also indicated that statin use in patients aged 8–18 with familial hypercholesterolemia (FH) could slow the progression of atherosclerosis, as well as reduce the risk of cardiovascular events and death [107, 108].

#### 5.3.3 COVID-19 Patients

During the Coronavirus Disease 2019 (COVID-19) pandemic, many infected individuals were taking statins. Multiple studies have explored whether the use of statins affects the prognosis of COVID-19 patients [109]. In a retrospective meta-analyses, statins were shown to reduce the mortality rate of COVID-19 patients by 31%, while RCT meta-analyses showed no significant reduction in mortality [109]. In conclusion, it is safe for COVID-19 patients to use statins, but whether it has any clinical benefits requires further evidence.

#### 5.3.4 Patients with Heart Failure and Atrial Fibrillation

The CORONA study [110] is the first large-scale RCT exploring the benefits of statins in patients with heart failure. Although the cardiovascular combined endpoint was negative, the risk of hospitalizations for cardiovascular causes was greatly reduced [110]. A meta-analysis of 17 studies showed that statin use in heart failure patients significantly lowered the risk of all-cause mortality and cardiovascular-related hospitalization [111]. In addition, patients with atrial fibrillation, cardioembolic stroke, and immune-mediated inflammatory diseases face increased risk of cardiovascular events [112, 113, 114]. These populations can use statins to decrease the risk of ASCVD and improve prognosis, as demonstrated in a meta-analysis, although most of the included studies were real-world studies [112, 113, 114].

## 6. Statins as Cornerstone Drugs for Combination Therapy in a Coming “Statins Plus” Era

**Fig. 6.**
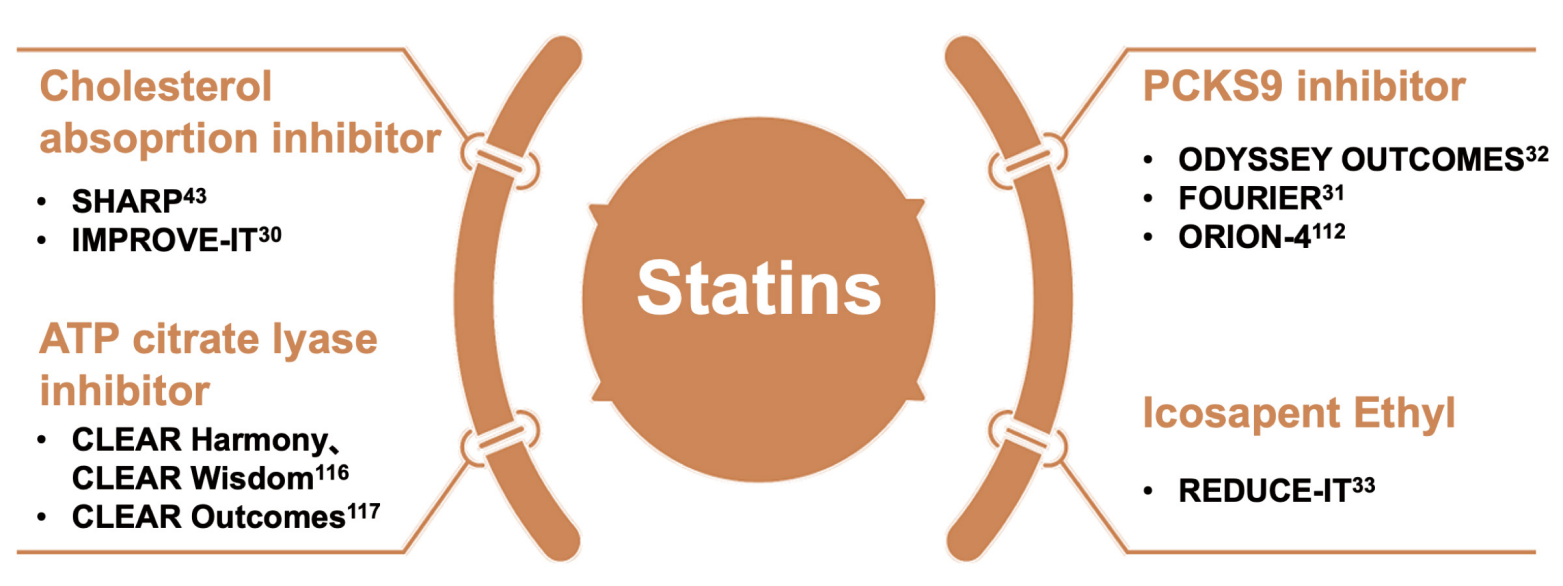
**Statins as cornerstone drugs for combination therapy**. ATP, adenosine triphosphate; PCSK9, proprotein convertase subtilisin/kexin type 9.

Recent advancements in cardiovascular pharmacotherapy have introduced a variety of new drugs targeting different aspects of lipid metabolism. Ezetimibe is a cholesterol absorption inhibitor that specifically targets LDL-C [30, 43]. Proprotein convertase subtilisin/kexin type 9 (PCSK9) inhibitors and adenosine triphosphate citrate lyase inhibitors like bempedoic acid, represent other innovative approaches [31, 32, 115]. Additionally, new peroxisome proliferator-activated receptor α (PPARα) agonists target triglyceride (TG), and omega-3 fatty acids, among others [33, 116]. Most of the cardiovascular outcome studies of these drugs used statin-based combination therapy in the intervention groups (Fig. 6).

### 6.1 Combination Therapy of Statins and Drugs Targeting LDL-C

#### 6.1.1 Statins Combined with Ezetimibe

Statins are complementary with ezetimibe [30, 43, 117]. When combined, they can further reduce LDL-C, slow down the progression of atherosclerosis, and lower the risk of cardiovascular events [30, 43, 117]. The Study of Heart and Renal Protection (SHARP) study showed that compared to placebo, the combination of statins and ezetimibe could significantly reduce the risk of cardiovascular events in patients with chronic renal disease [43]. The Improved Reduction of Outcomes: Vytorin Efficacy International Trial (IMPROVE-IT) study demonstrated that when added to statin therapy, ezetimibe resulted in incremental lowering of the risk of MACE in ACS patients [30].

#### 6.1.2 Statins Combined with PCSK9 Inhibitors

PCSK9 inhibitors reduce the degradation of LDL receptors, promote the clearance of LDL-C, and lower LDL-C levels [31, 32]. Currently available drugs include PCSK9 monoclonal antibodies, such as evolocumab and alirocumab, and the PCSK9 small interfering RNA inclisiran [31, 32]. The cardiovascular benefits of statins combined with PCSK9 monoclonal antibodies have been demonstrated in studies such as Further Cardiovascular Outcomes Research with PCSK9 Inhibition in Subjects with Elevated Risk (FOURIER) and Outcomes After an Acute Coronary Syndrome During Treatment With Alirocumab (ODYSSEY OUTCOMES) [31, 32]. These two studies added PCSK9 inhibitors to high-intensity or moderate-intensity statin therapy in ASCVD patients, and showed significant declines in cardiovascular endpoints and mortality [31, 32]. This once again proved the cholesterol theory and promoted the lowering of LDL-C target values in guidelines [31, 32].

Inclisiran has similar LDL-C lowering effects as PCSK9 monoclonal antibodies, but with a longer duration, and has been approved in many countries [118]. The cardiovascular outcome study the Effects of Inclisiran on Clinical Outcomes Among People With Cardiovascular Disease 4 (ORION-4) will combine inclisiran or placebo to high-intensity statin therapy in 15,000 ASCVD patients, and is expected to be completed in 2026 [115]. Currently, there are no large-scale cardiovascular outcome studies focusing exclusively on PCSK9 inhibitors.

#### 6.1.3 Statins Combined with Bempedoic Acid

Both bempedoic acid and statins target the cholesterol synthesis pathway, but act on different enzymes [119, 120]. Bempedoic acid targets ATP-citrate lyase (ACLY), an enzyme upstream of HMG-COA reductase [119, 120]. When used alongside statins, bempedoic acid has been shown to reduce LDL-C by about 20% when combined with statins [119, 120]. The recently published cardiovascular study Cholesterol Lowering via Bempedoic Acid, an ACL-Inhibiting Regimen (CLEAR) Outcomes study, which focused on patients intolerant to statins, found that bempedoic acid reduced the risk of MACE by 13% compared to placebo, with no significant impact on the risk of stroke or all-cause mortality [121].

### 6.2 Statins Combined with Targeting TG

#### 6.2.1 Statins Combined with Fibrates

Pemafibrate, the novel PPARα agonist, lowers TG levels by regulating the expression of PPARα and has been approved for the treatment of hypertriglyceridemia [116]. The recent cardiovascular outcome study PROMINENT showed that when combined with statins, Pemafibrate could significantly lower TG levels in patients with Type 2 diabetes, but there was no significant decline in the risk of cardiovascular events and death [116].

#### 6.2.2 Statins Combined with Omega-3 Fatty Acids

Omega-3 fatty acids, another TG-lowering treatment, can further reduce the risk of MACE when combined with Icosapent Ethyl (IPE), as demonstrated by the Reduction of Cardiovascular Events with Icosapent Ethyl–Intervention Trial (REDUCE-IT) trial [33]. However, the subsequent Epanova in High Cardiovascular Risk Patients with Hypertriglyceridemia (STRENGTH) trial (using omega-3 carboxylic acid) and OMEMI trial (combining eicosapentaenoic acid [EPA] with docosahexaenoic acid [DHA]) once again showed no cardiovascular benefits [122]. Therefore, statins should still be the cornerstone for patients with elevated TG, supplemented by fibrates and omega-3 fatty acids, unless TG is ≥500 and the risk of ASCVD is low [123]. In summary, while these drugs provide more options for blood lipid management, they still play a role when combined with statins.

## 7. Statins as Cornerstone Drugs due to Safety in Different Populations

### 7.1 Common Safety Concerns of Statins

In clinical practice statins have been widely used for the prevention and treatment of ASCVD. However their safety remains a concern, particularly potential impacts on the liver, muscles, kidneys, and the possibility of secondary-onset diabetes [124].

#### 7.1.1 Hepatic Safety

The main impact of statins on the liver is isolated elevation of transaminases, which are usually transient and asymptomatic [124]. The incidence of transaminase levels exceeding three times the upper limit of normal (ULN) is about 1%, while that of severe liver damage is about 0.001% [124]. Even in patients with underlying liver diseases such as non-alcoholic fatty liver or chronic hepatitis C, statins do not significantly increase the risk of liver injury [125, 126]. Currently, routine liver function tests are not required for statin users.

#### 7.1.2 Muscle Safety

Statins-associated muscle symptoms (SAMS) include myalgia, myositis, myopathy, and rhabdomyolysis. Overall, the incidence of SAMS does not exceed 1%, while the incidence of severe muscle injury is below 0.1% [124]. Rhabdomyolysis is the most severe symptom, but is extremely rare [124]. Although the incidence of SAMS remains rather high in observational studies, in RCT studies reveal a different picture. In these trials, the incidence of SAMS in the statin group shows either no significant difference or only a slight increase in the incidence of SAMS compared to the placebo group [127, 128]. Studies such as SAMSON suggest that SAMS is mainly due to the nocebo effect instead of the use of statins [129].

#### 7.1.3 Renal Safety

Although 40 mg of rosuvastatin might cause transient proteinuria and microscopic hematuria, it does not affect renal function [124]. A meta-analysis has shown that statin therapy could significantly reduce urinary albumin and slightly improve creatinine clearance [130]. Overall, statins, including rosuvastatin, do not damage renal function, nor do they cause acute renal failure unrelated to myopathy.

#### 7.1.4 New-Onset Diabetes

The increased risk of new-onset diabetes with statins is a class effect, and the mechanism is not yet clear. The incidence is approximately 0.2% per year and is mainly observed in patients with multiple risk factors for diabetes [124, 131]. Statins have a mild effect on hemoglobin A1c (HbA1c), usually without clinical significance [124].

#### 7.1.5 Other Safety Concerns

There have been reports of statins increasing the risk of hemorrhagic stroke, but this has not been shown in recent meta-analyses. The extremely low incidence (5–10 cases among 10,000 patients receiving treatment for five years) is outweighed by the benefits of ischemic stroke prevention [132, 133]. Although some experts had concerns about whether statins affect cognitive function, subsequent large-scale studies and RCTs do not support the view that statins impact human cognitive function [124].

### 7.2 Long-Term Safety of Statins

**Fig. 7.**
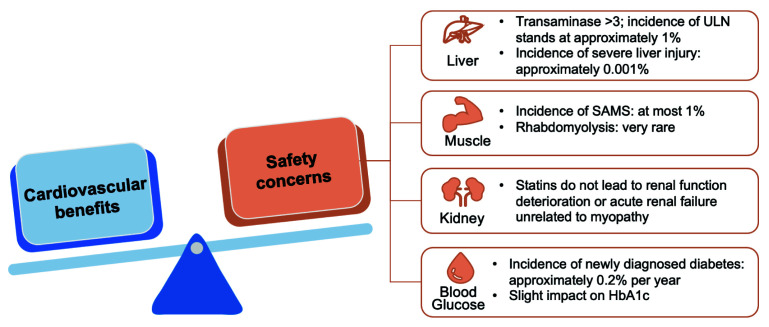
**The cardiovascular benefits of statins far outweigh the safety concerns**. ULN, upper limit of normal value; SAMS, statin-associated muscle symptom; HbA1c, hemoglobin A1c.

A systematic review of USPSTF, including 22 trials with follow-ups ranging from 6 months to 6 years, showed that statin therapy was not associated with a significantly increased risk of serious adverse events, myalgia, or liver-related injury [77]. Many classic RCTs on statins, with follow-ups of lasting at least 10 years, have demonstrated the long-term safety of these drugs. Notably, there was no apparent increase in the risk of mortality related to non-cardiovascular disease or cancer [134, 135, 136, 137]. These findings are consistent with the meta-analyses of trials with extended follow-up of more than six years [138]. In general, statins have good safety, and serious adverse events rarely occur. As pointed out by the AHA Scientific Statement: Statin Safety and Associated Adverse Events published in 2018, the cardiovascular benefits of statins far outweigh the safety concerns (Fig. 7) [124].

### 7.3 Safety of Statins in Special Populations

#### 7.3.1 Children and Adolescents

Dyslipidemia in children and adolescents has long been a severe problem, with a prevalence of about 20% [139, 140]. Studies have showed that statins can be safely used in children and adolescents, with a low incidence of adverse effects (AEs) and no effect on growth or sexual development [141].

#### 7.3.2 Pregnant Women

A recent meta-analysis showed no significant increase in rate of major congenital malformations and heart defects in pregnant women taking statins [142]. Therefore, in July, 2021, FDA requested removal of its strongest warning against using cholesterol-lowering statin drugs in pregnant patients, and suggested that medical professionals and patients should jointly evaluate the benefits and risks of statins use on a case-by case basis [143].

#### 7.3.3 Older Adults

When used in older adults, there is no significant difference in adverse events or incidence for the need to discontinue statins relative to the placebo group [144]. In the PROSPER trial, serious adverse events were reported with similar frequency in the statin-treated group and the placebo cohort among patients aged 70–82 [24]. Therefore, relevant guidelines recommend that older adults with ASCVD should use statins in the same manner as younger patients, and statins should also be considered for patients with high risk for ASCVD [145, 146].

#### 7.3.4 Patients with Renal Insufficiency

For patients with renal insufficiency, statins do not impact the decline of renal function; instead, they may even delay the process [124, 147]. Statin therapy can significantly reduce ASCVD risks for patients with mild to moderate renal insufficiency [124]. Studies have shown elevated levels of proteinuria among statin users, but this is transient, and there is no conclusive evidence on its causality with statin use [124]. However, statins are not recommended for non-ASCVD patients who are on dialysis [124].

## 8. Statins as Cornerstone Drugs Providing Diversified Options

**Fig. 8.**
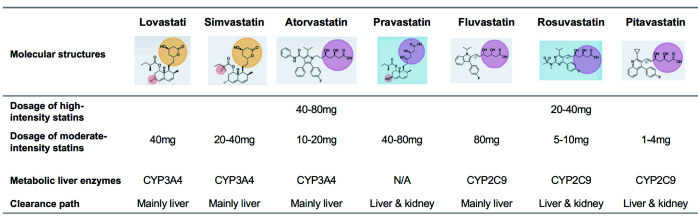
**Molecular structures and pharmacological effects of different types of statins**. The yellow circle area refers to actonic ring. The pink circle area refers to sour. The blue square refers to the hydrophilic. N/A, not applicable; CYP, cytochrome P450.

The cardiovascular benefits of several statins currently on the market have been confirmed (Fig. 5). Different types of statins, due to their different chemical structures, have their own characteristics in lipid-lowering and pharmacological effects, which can meet different therapeutic needs (Fig. 8) [124, 148]. The level of LDL-C decrease varies greatly among different types and dosing of statins. Based on the level of reduction, statins fall into high-intensity and moderate-intensity categories. In addition, the difference in statin metabolizing enzymes will also affect drug interactions, therefore in clinical practice, the types and dosage of statins should be selected according to patient co-morbidities.

In clinical practice, statins are the most widely used lipid-lowering drug with accessibility much higher than that of non-statins. In addition, statins have been included in the China National Essential Medicine List, and the lower price has greatly reduced patients’ economic burden for long-term use, so that more people can benefit from statins [149].

## 9. Statins as Cornerstone Drugs due to Its Comprehensive Effects on Lipid Profile

**Fig. 9.**
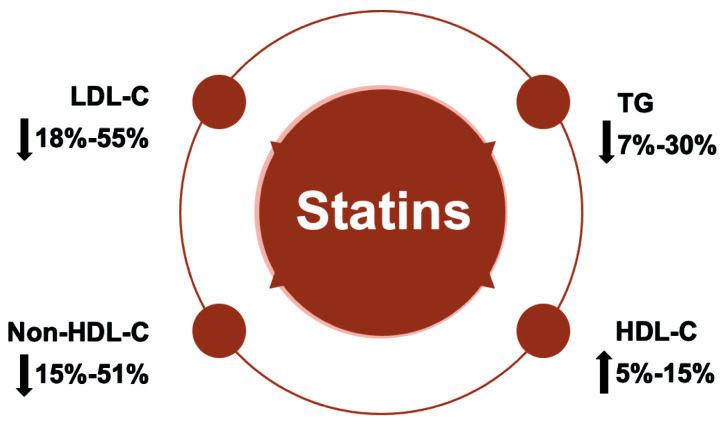
**Statins significantly improve blood lipid profile by lowering LDL-C, Non-HDL-C and TG levels and increasing HDL levels**. LDL-C, low-density lipoprotein cholesterol; HDL-C, high-density lipoprotein cholesterol; TG, triglyceride.

Research indicates that statins can significantly impact lipid profiles. Particularly, reducing LDL-C by 18%–55%, non-high density lipoprotein cholesterol (HDL-C) by 15%–51%, TG by 7%–30% and increase HDL-C by 5%–15% (Fig. 9) [150]. A number of trials suggested a link between elevated TG and an increased risk of ASCVD, and that statins could result in a dose-dependent reduction in TG [151].

Lipoprotein (a) (Lp(a)) has attracted much attention in recent years and has become a potential target for blood lipid management [152, 153, 154]. For patients with elevated Lp(a), the treatment should follow the principle of reducing the overall risk of ASCVD and managing other clinically significant dyslipidemias [152, 153, 155]. The AHA Statement suggests that it is reasonable to give moderate to high intensity statin therapy to patients with high Lp(a) [152]. A meta-analysis showed that statin therapy had no significant impact on Lp(a) [156], and that active statin therapy could reduce high ASCVD risk caused by Lp(a).

## 10. Statins as Cornerstone Drugs Recommended by Guidelines at Home and Abroad

Numerous evidence-based guidelines have made statins the cornerstone of drug therapy. Over the years, with the continuous update of blood lipid guidelines, statins have remained the top choice for lipid-lowering drugs recommended by guidelines at home and abroad, and are widely used in primary and secondary prevention of ASCVD [145, 146, 154].

The recently released Chinese Guidelines for Lipid Management (2023) continue to emphasize statins as the cornerstone of lipid-lowering treatment for dyslipidemia, with moderate-intensity statins recommended as the first choice for lipid-lowering therapy in China’s population [154]. The 2018 AHA/ACC Guideline on the Management of Blood Cholesterol suggests adding a PCSK9 inhibitor in patients at very high LDL-C risk already on maximal statin and ezetimibe therapy [145]. Similarly, the 2019 ESC/EAS Guidelines for the management of dyslipidemias recommends the highest tolerated statin dose to achieve a certain LDL-C target [146]. If the LDL-C target is not reached, statins may be used in combination with ezetimibe [146]. If the LDL-C target is not reached with the highest tolerated statin dose and/or ezetimibe, PCSK9 inhibitors may be considered in addition to LLT [146].

## 11. Conclusions and Perspectives

Statins have been available in the market for over 30 years. Since the 4S in 1994, the world has entered a new era of statin therapy. Many cardiovascular outcome trials on various types of statins have been carried out in a large number of patients. Such trails cemented the foundational role of statins in cholesterol lowering as well as primary and secondary prevention of ASCVD, and ushered in a new era of blood lipid management and ASCVD prevention. Emerging new lipid-lowering drugs still rely on combination therapies with statins as the cornerstone for certain clinical benefits (Fig. 3 and Table 1). In addition, despite statins being the cornerstone of lipid lowering therapy, there is a proportion of patients with high cardiovascular risk and atherosclerotic burden who cannot be managed solely on statins and would be best combined with non-statin lipid lowering treatments.

There are currently still many unsolved issues regarding statins, such as the impacts of statins on immune regulation, statin exposure and tumor risk, the mechanism of statin muscle-related adverse events, the efficacy and the safety of statins in special populations. In the future, patients suitable for statin therapy will be better identified, thereby allowing for more precise statin treatment for patient populations with the highest risk for ASCVD.

## Abbreviations

ACS, acute coronary syndrome; AMI, acute myocardial infarction; ASCVD, atherosclerotic cardiovascular disease; CABG, coronary artery bypass graft; CAD, coronary artery disease; CHD, coronary heart disease; CVD, cardiovascular disease; HDL-C, high-density lipoprotein cholesterol; HMG-CoA, 3-hydroxy-3-methyl-glutaryl-CoA; LDL-C, low-density lipoprotein cholesterol; LLT, lipid-lowering therapy; Lp(a), lipoprotein (a); MACE, major adverse cardiac event; MI, myocardial infarction; PCI, percutaneous coronary intervention; PCSK9, proprotein convertase subtilisin/kexin type 9; TG, triglyceride.
